# Transcript profiling of two potato cultivars during glycoalkaloid-inducing treatments shows differential expression of genes in sterol and glycoalkaloid metabolism

**DOI:** 10.1038/srep43268

**Published:** 2017-03-03

**Authors:** Nurun Nahar, Erik Westerberg, Usman Arif, Alexandre Huchelmann, Alexandra Olarte Guasca, Lisa Beste, Kerstin Dalman, Paresh C. Dutta, Lisbeth Jonsson, Folke Sitbon

**Affiliations:** 1 Department of Plant Biology, Uppsala BioCenter, Swedish University of Agricultural Sciences, and Linnean Centre for Plant Biology, P. O. Box 7080, 75007 Uppsala, Sweden; 2 Department of Food Science, Uppsala BioCenter, Swedish University of Agricultural Sciences, P. O. Box 7051, 75007 Uppsala, Sweden; 3 Department of Ecology, Environment and Plant Sciences, Stockholm University, 10691 Stockholm, Sweden

## Abstract

Steroidal glycoalkaloids (SGA) are sterol-derived neurotoxic defence substances present in several members of the Solanaceae. In the potato (*Solanum tuberosum*), high SGA levels may render tubers harmful for consumption. Tuber SGA levels depend on genetic factors, and can increase as a response to certain stresses and environmental conditions. To identify genes underlying the cultivar variation in tuber SGA levels, we investigated two potato cultivars differing in their SGA accumulation during wounding or light exposure; two known SGA-inducing treatments. Using microarray analysis coupled to sterol and SGA quantifications, we identified a small number of differentially expressed genes that were associated with increased SGA levels. Two of these genes, encoding distinct types of sterol Δ^24^-reductases, were by sense/antisense expression in transgenic potato plants shown to have differing roles in sterol and SGA metabolism. The results show that an increased SGA level in potato tubers during both wounding and light exposure is mediated by coordinated expression of a set of key genes in isoprenoid and steroid metabolism, and suggest that differences in this expression underlie cultivar variations in SGA levels. These results may find use within potato breeding and quality assessment.

The potato (*Solanum tuberosum*) has for long had a central role in human nutrition, and is today the fourth most important crop globally. However, in addition to desirable nutrients such as starch and vitamin C, potato tubers also contain toxic substances, among which the steroidal glycoalkaloids (SGAs) are the most important. SGAs are neurotoxic compounds that are present in several species within the Solanaceae, including crop species such as eggplant (*Solanum melongea*), tomato (*Solanum lycopersicum*), and potato^1^,^2^,^3^,^4^,^5^. Mild symptoms of glycoalkaloid toxicity include headache, nausea, and diarrhea, but consumption of potatoes with high SGA levels has also caused more severe poisonings^6^. For safety reasons, an upper limit of 200 mg total SGA kg^−1^ fresh weight (f.w.) is widely recommended in tubers used for human consumption. However, it is not unusual that this level is surpassed, and potato cultivars such as Lenape and Magnum Bonum have been withdrawn from the US and Swedish markets, respectively, due to SGA levels exceeding the recommended limit^7^,^8^. An increased understanding of the SGA biosynthetic pathway and its regulation in potato plants is hence of broad interest.

SGAs contain a steroidal skeleton to which sugars and sometimes additional molecules are bound, and over 80 different SGAs have been identified in potato^5^. In cultivated potato varieties, however, over 95% of the SGA content is accounted for by only two forms; α-chaconine and α-solanine. These are derived from the same aglycone, solanidine, but differ in their sugar moieties. SGAs occur in all parts of the plant, generally attaining the highest levels in flowers and berries. The tuber SGA level is lower, but can be increased several-fold by certain stresses and environmental conditions, such as mechanical damage and light exposure^1^,^9^. We have previously shown that not only is the basal SGA level varying between cultivars, but also the tendency for increased SGA upon wounding or light exposure^10^. For instance, King Edward displayed a strong response to both light and wounding, whereas Bintje showed significantly weaker responses, particularly to light.

SGAs are derived from the isoprenoid pathway, and the sterol cholesterol has been identified as a metabolic precursor^11^,^12^,^13^ (Supplementary Fig. S1). The biosynthetic pathway to cholesterol in plants is less understood than in humans, probably because cholesterol is a minor sterol in most plant species^14^,^15^,^16^. However, in potato and some other species such as tobacco (*Nicotiana tabacum*), cholesterol is a major sterol, accounting for between 5% and 20% of the 4-desmethyl sterols^17^,^18^, and it has also been shown to be the major sterol in phloem exudate in tobacco and Chinese cabbage (*Brassica rapa*)^19^. The formation of the cholesterol side-chain was recently investigated in potato^20^. The sterol Δ^24^-reductase SSR1 expressed in yeast converted 24-methylene cholesterol to campesterol, whereas SSR2 converted cycloartenol to cycloartanol, and desmosterol to cholesterol. Moreover, *SSR2*-silenced potato transformants displayed reduced levels of cholesterol, sitosterol, and SGA, in line with a role in sterol and SGA metabolism.

The understanding of how cholesterol is further metabolised to SGA has increased in recent years. Based on coexpression analyses of two genes relevant for biosynthesis of α-tomatine, the major SGA in tomato, a number of additional genes (denoted *GAME1* to *GAME18*) in α-tomatine biosynthesis were identified and functionally characterized^21^. Orthologous genes in potato were also described, leading to a model for SGA biosynthesis in potato and tomato^22^. Recently, the transcription factor GAME9 (also called JRE4) was shown to regulate the expression in tomato and potato of genes in the isoprenoid and SGA biosynthesis pathways^23^,^24^.

To identify genes important to the between-cultivar variation of SGA levels in potato, we have here taken a functional approach starting from the different SGA responses in cultivars Bintje and King Edward^10^. We hypothesized that genes relevant for increased SGA biosynthesis would fulfil at least three criteria: (i) be up-regulated by both wounding and light; (ii) be more strongly induced in King Edward than in Bintje; and (iii) be induced before, or parallel to, increased SGA levels. Our analyses show that SGA accumulation in potato tubers during wounding and light exposure is associated with a coordinated expression of a small number of key genes in isoprenoid and steroid metabolism, and suggest that this expression also underlies cultivar differences in SGA levels.

## Results

### Increased SGA levels in potato tubers are preceded by a cholesterol increase

**Figure 1:**
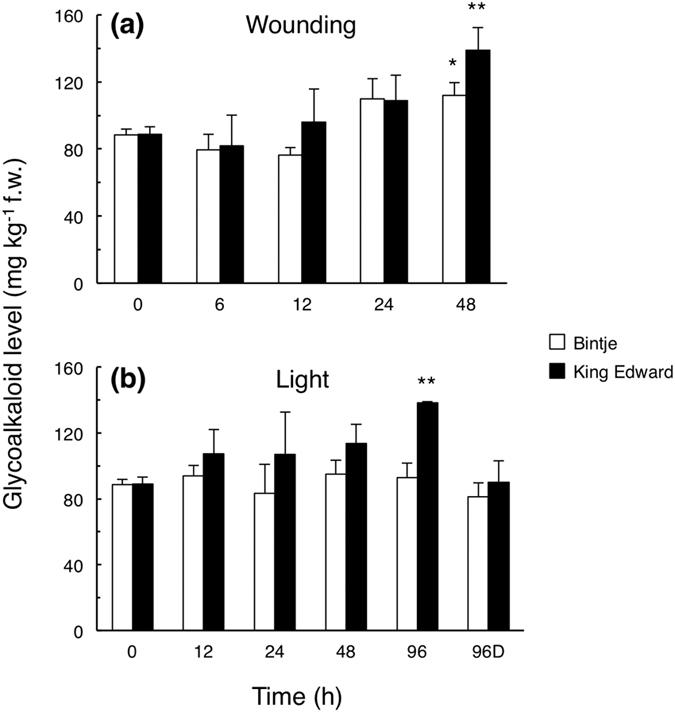
Glycoalkaloid levels in potato tubers subjected to wounding or a light exposure. Glycoalkaloid levels in tuber cross-sections were analysed at different time points after wounding (**a**), or continuous white light exposure (**b**), in Bintje (white bars) and King Edward (dark bars). 96D indicates a control kept 96 h in darkness. Mean glycoalkaloid value ± range from the sum of α-chaconine and α-solanine, as determined by HPLC-UV in biological duplicates for each cultivar. Asterisks indicate a regression analysis (ANOVA) of the cultivar response, significantly different from zero at p < 0.05 (*) or at p < 0.01 (**).

Within 48 h after tuber wounding, there was an increase in SGA levels in both King Edward and Bintje, being more significant in King Edward (Fig. 1a). Upon light exposure, the SGA level in King Edward tubers became clearly increased, whereas there was no SGA increase in Bintje tubers within the experimental time period (Fig. 1b). In control tubers kept in the dark, there were no changes in SGA contents (Fig. 1b). These results demonstrate a stronger SGA response in King Edward than in Bintje, both after wounding and during light exposure, which is well in accordance with our previous analyses of these genotypes^10^.

During the light exposure of tubers, a greener colour in King Edward than in Bintje became obvious by the eye (Supplementary Fig. S2). Determination of chlorophyll levels in tuber peels confirmed this observation: 99 ± 20 mg kg^−1 ^f.w. in King Edward, and 31 ± 5 mg kg^−1 ^f.w. in Bintje (mean ± s.d.; *n* = 3). Although chlorophyll and SGA synthesis are metabolically independent processes^25^, the results showed that also chlorophyll synthesis was more pronounced in King Edward than in Bintje.

**Figure 2:**
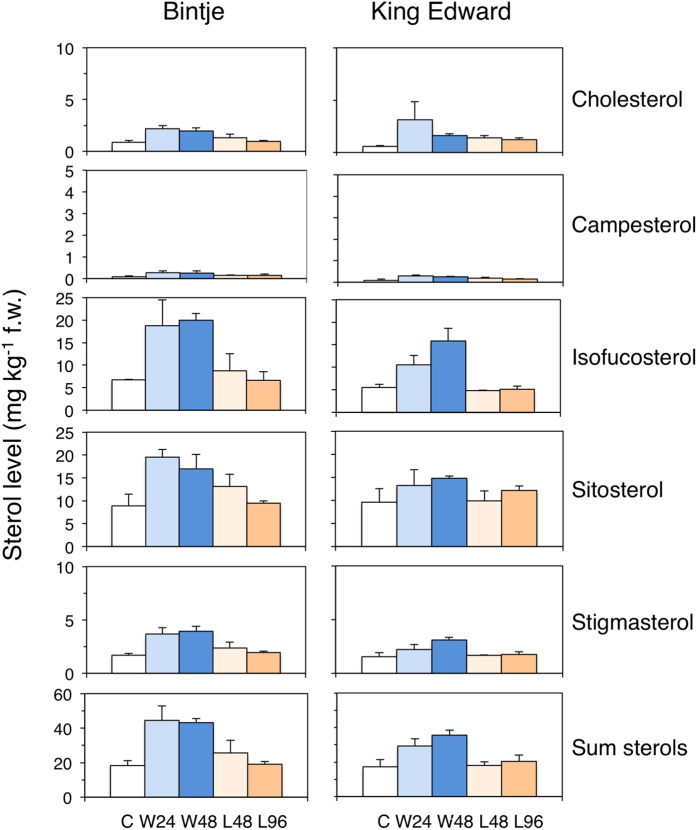
Sterol levels in potato tubers subjected to wounding or a light exposure. Free 4-desmethyl sterols were analysed in tuber cross-sections at the indicated time points after wounding (W) (blue staples; 24 h and 48 h), or a light exposure (L) (yellow staples; 48 h and 96 h), in Bintje and King Edward. Control samples (C) were sampled before treatment. Mean value ± range of two biological replicates per genotype consisting of three pooled tubers per sample, each of which was extracted and analysed in triplicate. For both cultivars, an increased total sterol level was significant after wounding, but not after light exposure (one-way ANOVA; p < 0.05).

Sterol levels were analysed to investigate parallel changes in SGA precursors (Supplementary Fig. S3). For both cultivars, wounding resulted in an increase in the level of all major 4-desmethyl sterols (Fig. 2). The cholesterol level increased in both cultivars within 24 h; about 2-fold in Bintje, and between 3- and 5-fold in King Edward. The proportion of cholesterol to total 4-desmethyl sterols was during this period constant at 5% in Bintje, but increased from 3% to 8% in King Edward.

Light exposure led within 96 h to a significant 2-fold cholesterol increase in King Edward (p < 0.05; *t*-test), whereas the corresponding changes in Bintje were less pronounced and statistically insignificant (Fig. 2). The level of other 4-desmethyl sterols were not significantly altered by light in any cultivar. Thus, for both types of treatment, there was a cholesterol accumulation preceding, or parallel to, the increased SGA. This suggests that SGA accumulation in potato tubers at least partly is regulated before cholesterol.

### Transcript profiling reveals distinct cultivar responses to wounding or light exposure

**Figure 3:**
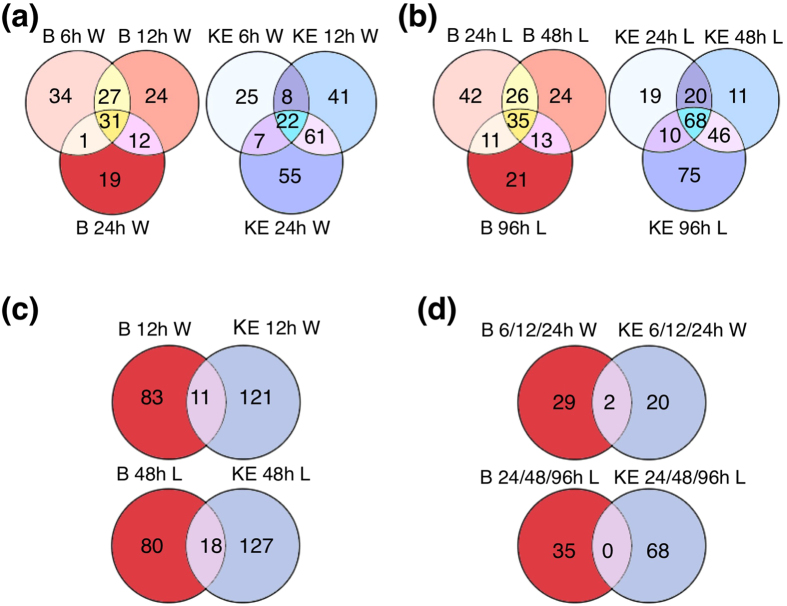
Numbers of differentially expressed genes in potato tubers subjected to wounding or a light exposure. (**a**) Venn-diagram display of differentially expressed genes in Bintje (B) and King Edward (KE) tubers at 6 h, 12 h, and 24 h after wounding (W). (**b**) Differentially expressed genes in Bintje and King Edward tubers at 24 h, 48 h, and 96 h from onset of a light exposure (L). (**c**) Differentially expressed genes in Bintje and King Edward at 12 h after wounding, or at 48 h from onset of a light exposure. (**d**) Genes differentially expressed at all three time points investigated after wounding, or from onset of a light exposure. For all treatments, a differential expression was measured in comparison to the experimental start point (0 h). Differential expression was defined using B-statistics with a p-value threshold of p < 0.0001.

To identify genes underlying the increased tuber SGA levels, transcriptome changes during the wounding and light exposure treatments were compared in Bintje and King Edward. In both cultivars, the gene expression was significantly altered after wounding and light exposure (Supplementary Tables S1–S4). Changes were detectable already at the earliest time points analysed; *i.e*. at 6 h after wounding, and at 12 h after onset of light exposure. For both treatments, the number of significantly altered transcripts tended to increase with time in King Edward, but to decrease in Bintje (Fig. 3a,b). In intact King Edward control tubers kept in darkness, only 16 genes altered their transcript abundance after 96 h, whereas 199 genes were significantly altered after 96 h of light exposure, and 162 genes at 48 h after wounding (Supplementary Table S5). This shows that the majority of changes in gene expression were due to the respective treatments.

The identity of genes showing altered transcript abundance upon the two types of treatment differed between the two cultivars at all time points analysed. As examples, only 11 significantly regulated genes overlapped between the cultivars at 12 h after wounding, and only 18 genes at 48 h after light exposure (Fig. 3c). The between-cultivar difference was obvious also when comparing genes whose expression was significantly altered at all time points during the experiments. Only 2 genes were here overlapping between the cultivars after wounding, and none after light exposure (Fig. 3d).

To further characterize the changes in gene expression, genes were classified functionally by gene ontology (GO) annotation, and their kinetic response profile was categorised using the Short Time-series Expression Miner (STEM) software^26^. Each EST expression pattern is here correlated to a specific temporal expression among 50 model templates that are defined independently of the input data. Profiles with a statistically higher number of ESTs are identified from a permutation test based on the time points. The statistically significant profile patterns are depicted in Supplementary Fig. S4, and full lists are given in Supplementary Tables S6–S9.

After wounding, genes with a role in biogenesis of cellular structures (particularly the cell wall), metabolism, and stress responses, were overrepresented in both genotypes among the genes that were assigned to significant temporal response patterns by the STEM software (Supplementary Fig. S5a). In addition, genes with a role in energy/electron transport were overrepresented specifically in Bintje, and genes in defence processes overrepresented specifically in King Edward. After light exposure, genes in photosynthesis and stress responses were overrepresented in both genotypes, and genes in metabolism specifically in King Edward (Supplementary Fig. S5b).

A closer examination of genes in the ‘metabolism’ class showed for both genotypes that genes with a role in the metabolism of amino acids and of secondary products were overrepresented among the genes showing a significant response profile after wounding (Supplementary Fig. S5c). Moreover, an overrepresentation of genes in sterol metabolism was significant in King Edward, whereas genes in hormone metabolism/action were significant in Bintje. Light exposure led in both genotypes to an overrepresentation of genes in secondary metabolism and hormone metabolism/action (Supplementary Fig. S5d). In King Edward, also genes in chlorophyll biosynthesis and sterol metabolism were overrepresented.

The gene profiling results agreed well with the metabolic analyses, *e.g*. higher levels of chlorophyll and cholesterol after light exposure in King Edward compared to Bintje. Furthermore, genes related to photosynthesis were overrepresented only in the light exposure experiment. This demonstrates a capacity of STEM analysis to detect biologically relevant gene expression patterns, and supports the relevance of other significant expression patterns identified.

### Identification of key genes associated with increased SGA levels

**Figure 4:**
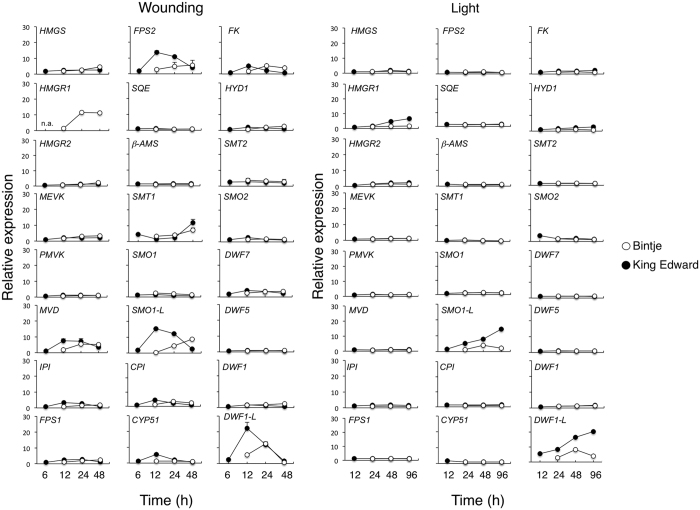
Temporal expression of sterol-related genes in potato tubers subjected to wounding or a light exposure. Microarray analysis of potato tubers from Bintje (open circles) and King Edward (dark circles), subjected to wounding (left), or a light exposure (right). Average expression values from duplicate measurements of two biological replicates ± range or s.d. of related ESTs where applicable. Potato orthologues to *Arabidopsis thaliana* sterol-related genes were identified by BLAST analysis. Scores and gene abbreviations are given in Supplementary Table S10. n.a; not analysed.

To pinpoint genes mediating the altered sterol and SGA metabolism during wounding and light exposure, we monitored the expression of the 24 potato orthologues to known *Arabidopsis thaliana* isoprenoid- and sterol-biosynthesis genes that were present on the microarray (Supplementary Table S10). After wounding, 19 of these genes displayed an expression pattern that was similar in Bintje and in King Edward (Fig. 4). Hence, these genes are not expected to account for any major differences between the cultivars in their wound-regulated increase of sterols or SGA. Among such genes were *SQE* (Squalene epoxidase), *SMO1* (Sterol C4-methyl oxidase type-1), and *DWF1* (Sterol Δ^24^-reductase). On the other hand, four genes showed a stronger and more rapid wound-induction in King Edward, and are thus more likely to be relevant for the stronger sterol and SGA response in that cultivar. These genes included *MVD* (Mevalonate diphosphate decarboxylase), *FPS2* (Farnesyl diphosphate synthase 2), *SMO1-LIKE* (Sterol C4-methyl oxidase 1-like; denoted *SMO1-L*), and *DWF1-LIKE* (Sterol Δ^24^-reductase-like; denoted *DWF1-L*) (Fig. 4).

The expression profile of sterol-related genes after light exposure was similar to that after wounding, although *MVD* and *FPS2* were not upregulated in this case. Three genes: *HMGR1* (3-hydroxy-3-methylglutaryl-CoA reductase 1), *SMO1-L*, and *DWF1-L* were more strongly induced in King Edward than in Bintje (Fig. 4). An independent light exposure experiment of King Edward tubers confirmed that the transcript abundance of *DWF1-L* increased during conditions of increased SGA synthesis, whereas that of *DWF1* did not change (Supplementary Fig. S6).

**Figure 5:**
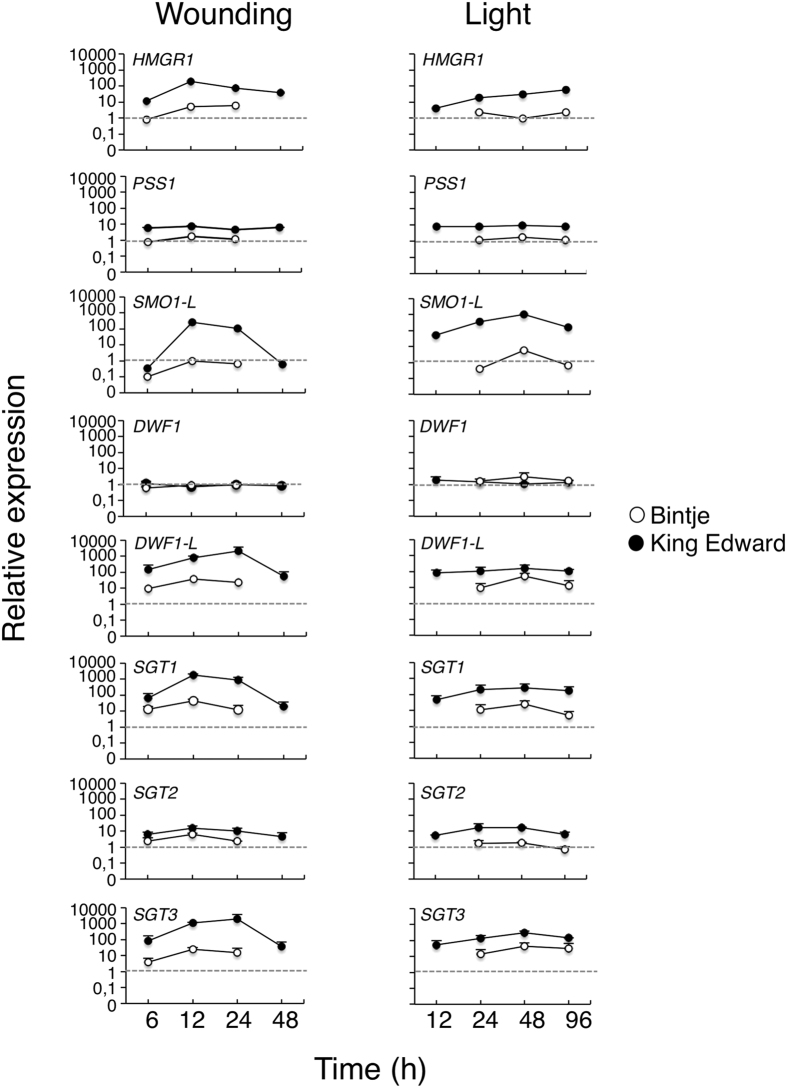
Quantitative real-time PCR analysis of gene expression in potato tubers subjected to wounding or a light exposure. Gene expression was analysed in tuber cross-sections by QPCR, normalized using potato β*-TUBULIN* as an internal reference, and expressed to the normalized expression level in non-treated samples (0 h). Average value ± range of two biological replicates from Bintje (open circles) and King Edward (dark circles) analysed in duplicate or triplicate. Gene abbreviations are given in Supplementary Table S10.

Quantitative real-time PCR (QPCR) was used to validate the induction of *HMGR1, SMO1-L*, and *DWF1-L* genes by both wound and light treatments, and a constitutive expression of *DWF1*. Four genes not represented in the microarray were also included, as they might be relevant for the SGA response. These genes encoded the potato squalene synthase PSS1^27^, and the final glycosylation enzymes SGT1 (solanidine galactosyl transferase), SGT2 (solanidine glucosyl transferase), and SGT3 (β-solanine/β-chaconine rhamnosyl transferase)^28^,^29^,^30^. The QPCR analysis confirmed the microarray data, showing that the abundance of *HMGR1, SMO1-L* and *DWF1-L* transcripts increased after the two treatments, and had a more rapid and stronger up-regulation in King Edward than in Bintje (Fig. 5). Further, the *SGT1* and *SGT3* gene expression profiles were similar to those of *HMGR1*/*SMO1-L*/*DWF1-L*, whereas *PSS1* and *SGT2* were less strongly induced. By contrast, *DWF1* was not up-regulated at any time point in the two cultivars.

To investigate if the observed *DWF1-L* expression differences between Bintje and King Edward could be attributed to promoter structures, 1300 bp of the *DWF1-L1* upstream sequence was amplified from both cultivars. However, the promoter DNA sequence was almost identical between the cultivars for at least this gene copy (Supplementary Fig. S7). More work is thus needed to determine if these minor sequence differences are functionally important, or if other factors are more relevant for the observed *DWF1-L* expression differences.

To identify additional genes with a putative role in the induction of SGA levels, we screened the King Edward array data and STEM profiles for genes coexpressed with *HMGR1*/*SMO1-L*/*DWF1-L*. This identified two cytochrome P450 (CYP450) genes; *CYP88B* and *CYP72A188* (Supplementary Table S11). There were over fifty CYP450 ESTs present on the array, including two additional *CYP72A* members (*CYP72A186* and *CYP72A208*), but *CYP72A188* and *CYP88B* were the only *CYP450* genes that were induced by both wounding and light treatments (Supplementary Fig. S8). A role for these genes in the cholesterol hydroxylation part of SGA biosynthesis has been shown by Itkin *et al*.^21^, who denoted the genes *GAME4 (CYP88B*) and *GAME6 (CYP72A188*). Also coexpressed with *HMGR1*/*SMO1-L*/*DWF1-L* were two other genes with a role in SGA biosynthesis (Supplementary Fig. S8). These genes encoded proteins highly similar to 2-oxoglutarate dioxygenase (2-OG; GAME11) and γ-amino butyric acid transaminase (TAM; GAME12), which contribute to the incorporation of nitrogen in the hydroxycholesterol molecule and subsequent F-ring closure^21^.

Together these results indicate that the increased SGA biosynthesis that occurs during both wounding and light exposure is mediated by coordinated expression of a small set of key genes that cover the entire SGA biosynthesis pathway; from the initial step catalysed by HMGR1, to the final step performed by SGT3.

### DWF1-L genes are duplicated in Solanaceous plant species containing SGA

The presence of two differentially regulated types of DWF1 genes in potato contrasted to the situation in *A. thaliana*, where the AtDWF1 protein is encoded by a single-copy gene (At3g19820). In the fully sequenced genome of the wild potato *Solanum phureja*, one gene of each type was revealed, both of which had a similar intron/exon organization (Supplementary Fig. S9). To investigate potato DWF1-encoding genes in more detail, full-length cDNA clones (cv. Kennebec) for one DWF1 and three DWF1-L cDNA were sequenced. Comparison of the deduced amino acid (aa) composition of DWF1 with that of either DWF1-L1, DWF1-L2 or DWF1-L3, showed ca. 79% aa identity over the entire protein, and revealed a characteristic insertion of 3 to 4 hydrophobic aa residues at the DWF1-L1/L2/L3 N-terminals (Supplementary Fig. S10). Both types of protein contained features characteristic for plant DWF1 proteins, including a putative transmembrane domain, a FAD-binding region, and a calmodulin-binding domain.

A phylogenetic analysis of full-length DWF1 proteins with emphasis on Solanaceous plant species, showed that the potato and tomato DWF1 proteins grouped together with DWF1 from *A. thaliana* and other plant species such as tobacco, rice and banana (*Musa acuminata*) (Supplementary Fig. S11). On the other hand, the DWF1-L type was present in potato, tomato, eggplant, pepper (*Capsicum annum*), and tobacco, *i.e*. plant species that contain SGA^31^, and formed a separate cluster. The occurrence of *DWF1*/*DWF1-L* duplications in SGA-containing plant species might provide these species with a unique means to simultaneously separate a need for housekeeping sterol biosynthesis, from the stress-induced use of sterols as precursors for defence metabolites such as SGA.

### Transgenic potato plants expressing *DWF1* genes in sense or antisense orientation display altered sterol profiles and SGA levels

The transcription profiling suggested a role for DWF1-L in the increased level of sterols and SGA after wounding and during light exposure. Functions of *DWF1-L* and *DWF1* were investigated in sense and antisense orientation in transgenic potato plants. These cDNAs were nearly identical to the potato sterol Δ^24^ -reductases SSR1 and SSR2^20^, and differences probably only reflect cloning from different cultivars.

Sense 35 S:*DWF1* and 35 S:*DWF1-L1* transformants (Supplementary Fig. S12) displayed an increased total sterol level (Supplementary Table S12). In particular, the sitosterol and stigmasterol levels were in both lines significantly higher than those in controls. However, there were no significant increases in cholesterol, or SGA levels, in leaves or tubers in the transformants (Table 1, Supplementary Table S12).

Antisense *asDWF1* clones showed a strong reduction of the *DWF1* transcript level in leaves, as determined by gel blot analysis of leaf RNA (Supplementary Fig. S13). The corresponding expression of *DWF1-L* was below detection in wild-type plants (not shown), so a QPCR screen was undertaken to identify *asDWF1-L* clones with reduced target gene expression. Extended QPCR analysis of *asDWF1* and *asDWF1-L* clones showed that the two antisense constructs were specific for their respective target gene (Supplementary Fig. S14).

**Figure 6:**
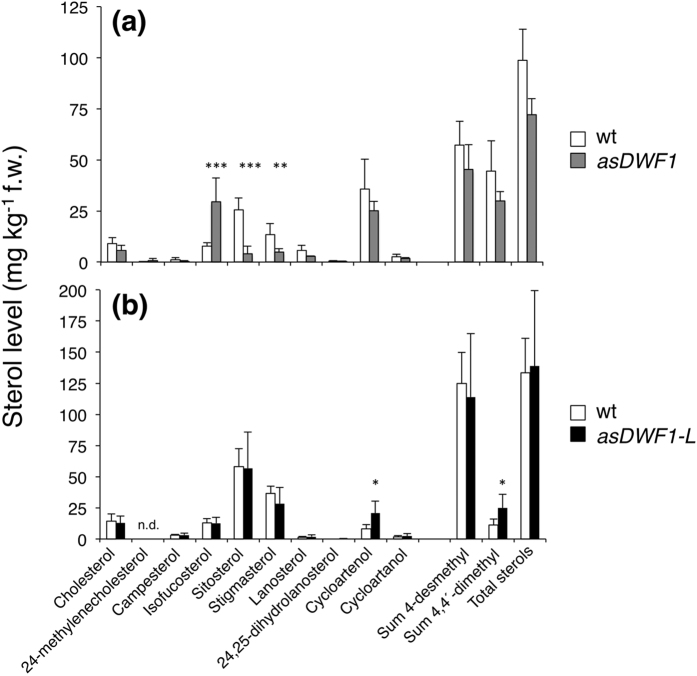
Leaf sterol composition in wild-type potato plants, and antisense *asDWF1* and *asDWF1-L* transformants. (**a**) Mean ± s.d. of leaf sterols extracted from wild-type Désirée plants (white bars; *n* = 8 plants), and the *asDWF1* clone #3 (grey bars; *n* = 6 plants). (**b**) Mean ± s.d. of wild-type plants (white bars; *n* = 6 plants), and the *asDWF1-L* clone #36 (dark bars; *n* = 6 plants). Asterisks indicate a difference from wild-type significant at p < 0.05 (*), p < 0.01 (**), or p < 0.001 (***) (Student´s *t*-test).

Sterol profiling in leaves of two *DWF1* antisense clones showed a strongly significant increase in the level of the DWF1 substrate isofucosterol^32^, and a corresponding decrease in its product sitosterol and further metabolite stigmasterol (Fig. 6a). Moreover, the ratio of 24-methylene cholesterol to campesterol increased from 0.15 in the wild type, to ca. 0.4 in transformants, supporting also 24-methylene cholesterol as a substrate, but here in campesterol formation^32^. The level of 4-monomethyl sterols was largely negligible, having a total sum below 5 μg kg^−1^ f.w. (not shown), and the profile of 4,4-dimethyl sterols was not significantly altered compared to the wild type (Fig. 6a). Thus, the *asDWF1* clones displayed an inhibition of sterol reductions at the side-chain ∆^24(28)^ position, leading to accumulation of the substrates isofucosterol and 24-methylene cholesterol. By contrast, *asDWF1-L* clones did not display any significant differences in the profile of leaf 4-desmethyl sterols compared to wild-type plants (Fig. 6b). The only significant difference was an increase of the 4,4-dimethyl sterol cycloartenol (Fig. 6b), a sterol having a ∆^24(25)^ double bond. The sterol profiles in *asDWF1* and *asDWF1-L* tubers were similar to those in the leaves (Supplementary Fig. S15), although the higher level of cycloartenol in *asDWF1*-L tubers was in this case not statistically significant.

**Figure 7:**
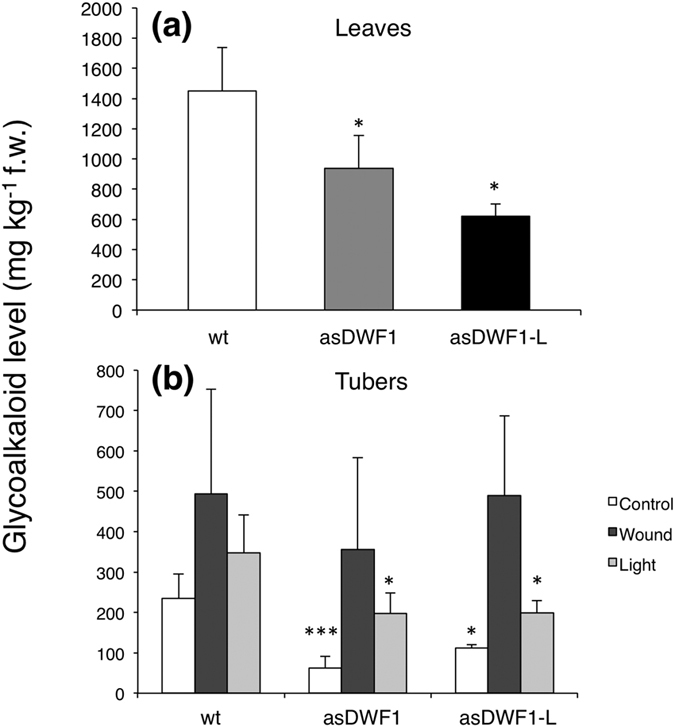
SGA levels in wild-type potato plants, and antisense *asDWF1* and *asDWF1-L* transformants. (**a**) SGA levels in leaves of wild-type cv. Désirée plants (*n* = 5), *asDWF* clone #3 (*n* = 4) and *asDWF1-L* clone #36 (*n* = 2). (**b**) SGA levels in un-treated control tubers, and in tubers exposed to wounding (2 d), or continuous white light (7 d). Mean ± s.d. or range, for analyses of wild-type, a*sDWF1* clone #3 (*n* = 5) and *asDWF1-L* clone #36 (*n* = 3). Sampling was performed at least at three separate occasions during a time period of more than one year. Each sample consisted of 2–3 pooled tubers from batches that had been harvested, stored and treated in parallel for each series of experiment. Asterisks indicate a difference from wild-type plants significant at p < 0.05 (*), or p < 0.001 (***) (Student´s *t*-test).

Compared to the wild type, SGA levels were lower both in leaves and tubers from *asDWF1* and *asDWF1-L* transformants (Fig. 7). In tubers exposed to wounding or to light exposure, both types of transformant responded by increasing SGA levels. The increased SGA level after light exposure was lower than the corresponding level in the wild-type, whereas SGA levels after wounding were more comparable (Fig. 7b). Despite their altered sterol profiles, the growth and development of both sense and antisense *DWF1* and *DWF1-L* clones was fully normal. The plant height at anthesis, number of tubers, and total tuber weight, did not differ significantly from wild-type controls (not shown).

## Discussion

### Transcript profiling reveals genes important to SGA accumulation

Earlier studies have shown that the SGA increase in potato tubers upon wounding or light exposure differs considerably between cultivars^10^. The present study confirms and extends such differences for cvs. Bintje and King Edward. Analyses of chlorophyll and sterols showed that not only the increase in SGA differed between these cultivars (Figs 1 and 2). Instead, the transcript profiling indicated that these results were only a minor part of metabolic differences, since there was little overlap between Bintje and King Edward in differentially expressed genes at any time point (Fig. 3).

The transcript profiling and QPCR analyses showed five sterol/SGA-related genes to be associated with increased SGA levels in response to wounding or light exposure; *HMGR1, SMO1-L, DWF1-L, SGT1*, and *SGT3* (Figs 4 and 5). Coregulated genes with a likely role in the post-cholesterol part of SGA biosynthesis were also identified in the same STEM clusters, including the genes *CYP88B, CYP72A188, TAM* and *2-OG* (Supplementary Fig. S8). For all treatments, a higher SGA level in King Edward than in Bintje was associated with a stronger expression of these genes. An important role for *HMGR1* in potato SGA biosynthesis has been indicated from up to 5-fold increases in leaf SGA levels in *HMGR1*-overexpressing potato transformants, whereas an effect of *SQS1* (here denoted *PSS1*) overexpression was less clear^27^. Our results agree also with those of Itkin *et al*.^21^, who showed that RNAi inhibition of *GAME4 (CYP88B*) expression in transgenic potato plants strongly inhibited SGA (α-solanine and α-chaconine) synthesis, and nullified the otherwise increased SGA production after light exposure. Likewise, RNAi inhibition of *GAME4* in tomato plants led to lower SGA levels (α-tomatine and esculeoside) and altered sterol profiles, whereas overexpression increased α-tomatine production. In addition, severely reduced α-tomatine levels were shown in tissues with virus-induced silencing of *GAME11 (2-OG*) and *GAME12 (TAM*), suggesting a role for the corresponding proteins in SGA synthesis^21^. Based on these studies, our results support that SGA accumulation in potato tubers is mediated by coordinated expression of a small set of key genes acting in the isoprenoid as well as steroid parts of the SGA-biosynthetic pathway, and also suggest that differences in this expression underlie the variation in SGA levels between potato cultivars.

Interestingly, a majority of the identified key genes (*e.g. DWF1-L, CYP72A188, 2-OG, TAM, SGT1, SGT3*) were identified in two recent studies as genes regulated by the transcriptional factor GAME9/JRE4; a regulator of gene expression in isoprenoid and SGA metabolism in potato and tomato^23^,^24^. This raises the possibility that GAME9/JRE4 is important to the cultivar-dependent gene regulation and SGA accumulation during wounding and light responses that we here have identified. In line with this is that GAME9 is likely identical to a major QTL associated with SGA content^24^, and that the QTL patterns of non-treated and light-exposed tubers are similar^33^.

### DWF1 and DWF1-L are involved in sterol and SGA biosynthesis

Reduction of the sterol side-chain in plants, humans and other organisms occur at the ∆^24(25)^ position, and is catalysed by sterol side-chain reductases. In case of plant ∆^24(28)^ sterols, e.g. isofucosterol and 24-methylene cholesterol, the conversion commonly includes an isomerisation to a ∆^24(25)^ intermediate that undergoes the reduction^14^,^32^. Our analyses revealed two types of sterol ∆^24^-reductase genes in potato; *DWF1* and *DWF1-L*, that clearly differed in their regulation by wounding or light exposure. Sterol profiling of the respective antisense transformants suggested DWF1 as a isofucosterol/24-methylene cholesterol reductase, but DWF1-L as a cycloartenol reductase (Fig. 6). The accumulation of isofucosterol and 24-methylene campesterol in *asDWF1* transformants is well in line with sterol profiles of *dwf1* mutants in *A. thaliana*, pea (*Pisum sativum*), and rice^32^,^34^,^35^. The accumulation of cycloartenol in *asDWF1-L* corroborates recent results from Sawai *et al*.^20^, who during the completion process of our manuscript showed by protein expression in yeast that that cycloartenol is a substrate for SSR2 (DWF1-L), and that potato *SSR2* RNAi transformants as well as plants with targeted *SSR2* gene disruption accumulate cycloartenol.

In keeping with a role for sterol ∆^24^-reductases in SGA synthesis was a reduced basal SGA level in both *asDWF1* and *asDWF1-L* transformants, and an attenuated SGA increase during light exposure (Fig. 7). However, an associated significant decrease in the SGA precursor cholesterol was not always observed. This contrasts to some extent to the results from Sawai *et al*.^20^, who reported on significantly reduced levels of cholesterol and SGA in *SSR2*-silenced plants. At present, we do not know the reason for these differing results, but variations in down-regulation efficiency or specificity are plausible explanations. It should be noted that neither the level of cholesterol (a C_27_ sterol), nor that of SGA was significantly affected in our *DWF1* and *DWF1-L* overexpressors, although the level of C_28_ and C_29_ sterols increased (Table 1 and Supplementary Table S12). This can for DWF1-L be explained if the enzyme to some extent is able to accept substrates in both the C_27_ and C_29_ sterol pathways. Increased channelling of unmetabolised precursors towards C_29_-sterol end products in overexpressors is also possible, and the result indicates that sterol ∆^24^-reductases alone are not limiting for SGA biosynthesis.

### Cholesterol biosynthesis in plants

Increased SGA levels in tubers were for both the wounding and light responses associated with an increased level of cholesterol, and an up-regulation of the sterol-biosynthetic genes *HMGR1, DWF1-L*, and *SMO1-L* (Figs 2 and 4). Related genes in *A. thaliana* have been identified from metabolite-to-gene studies as key genes in sterol synthesis. Overexpression of *HMGR* and *SMO1*, particularly in combination, clearly increased total sterol levels^36^. It is thus tempting to speculate that the concerted action of *HMGR1, DWF1-L* and *SMO1-L* in potato would be part of an activation of cholesterol biosynthesis. The biosynthetic pathway to cholesterol in plants is however poorly understood, and two alternative pathways are proposed in Supplementary Fig. S16. The first pathway has been suggested earlier, and implies that cycloartenol is a substrate for a sterol Δ^24^-reductase resulting in cycloartanol, which then is converted via C4-demethylation^37^,^38^. Support is derived from the increased levels of cycloartenol in *asDWF1-L* transformants but not in *asDWF1* ones (Fig. 6), as well as from the identification of cycloartanol and 31-norcycloartanol in potato slices^39^. An alternative conversion of cycloartenol would be an initial C4-demethylation to 31-norcycloartenol, which then would be converted via a Δ^24^-reductase to 31-norcycloartanol. This is to some extent supported by the identification of 31-norcycloartenol in potato slices^39^, and in bramble cells treated with 25-aza-cycloartanol^40^. Other steps in the model would be similar to the parallel pathways of C_28_ and C_29_ sterols in *A. thaliana*^15^, and are supported by the identification of some of the suggested intermediates (lophenol, 31-norlanosterol) in the present study.

Besides cycloartenol synthase, plants contain also lanosterol synthase^41^. Thus, analogous to the first pathway, also lanosterol might be reduced by a Δ^24^-reductase (to 24,25-dihydrolanosterol), and then undergo C4-demethylation. In an early experiment, radiolabelled lanosterol was in sorghum (*Sorghum bicolor*) reported to be more efficiently converted to cholesterol than to 24-methyl sterols or sitosterol^42^. This pathway would however need a SMO1 enzyme with different substrate specificity than that studied in maize, since this enzyme did not accept 24,25-dihydrolanosterol as substrate^43^. Further, the increase of cycloartenol levels without a concomitant lanosterol increase in our *asDWF1-L* transformants, indicates that lanosterol is not a preferred substrate at least for this specific sterol reductase. Moreover, results from studies utilising SMT1 inhibition^44^, the *A. thaliana smt1* mutant^45^, and *SMT1* overexpressors^18^, collectively suggest that also SMT1 activity will influence the flow of precursor metabolites towards either C_27_ sterols (cholesterol), or C_28_/C_29_ sterols.

## Conclusion

In summary, our results demonstrate that the SGA accumulation in potato tubers during both wounding and light exposure is mediated by coordinated expression of a small set of key genes covering important steps of the entire SGA-biosynthetic pathway, and suggest that differences in key gene expression also underlies the cultivar variation in basal SGA levels. The degree of SGA induction in a certain genotype is thus a trait that might become predictable based on screenings focussing on key gene expression. These findings open for new approaches in predicting the SGA response to environmental stress conditions, and may find use within potato breeding and quality assessment.

## Methods

### Plant materials and cDNA

Potato (*Solanum tuberosum* L.) tubers, cvs. Bintje and King Edward, were on the same day purchased from two separate sources for each cultivar. Tubers were synchronized by storage in darkness for three weeks at 8 °C, followed by two days at 20 °C. Care was taken to only include tubers of similar weight (60 g ± 5 g) and free of visible damage.

For generation of transgenic potato plants, full-length *DWF1* and *DWF1-L1* cDNA were PCR-amplified from potato cv. Kennebec cDNA clones provided by The Arizona Genomics Initiative, and fused in sense or antisense orientation to the CaMV 35 S promoter in Ti-plasmid pPCV702^46^. Potato cv. Désirée was transformed as described^47^. Nucleotide sequences of the *DWF1* and *DWF1-L* cDNA clones were deposited (30-June-2010) in the EMBL nucleotide database with accession numbers: StDWF1 (FN995649), StDWF1-L1 (FN995650), StDWF1-L2 (FN995651), StDWF1-L3 (FN995652).

### Tuber wounding and light exposure treatments

For wounding, the central part of a tuber was cut into two 5 mm thick transversal discs; one for RNA extraction and one for sterol analysis. For each biological replicate and time point, one such disc from each of three tubers was incubated in darkness at 20 °C between moist filter papers for either 0, 6, 12, 24, or 48 h, and then pooled into one sample for RNA extraction. This was repeated in parallel to give corresponding samples for sterol analyses. Remaining tuber parts, excluding tuber ends, were cut, incubated and pooled similarly for SGA analysis.

For light exposure experiments, intact tubers were incubated at 22 °C under constant white fluorescent light (110 μmol m^−2^ s^−1^) in a growth cabinet^10^, for 0, 12, 24, 48, or 96 h. As a control, tubers were kept 96 h in darkness. At the specified time points, three tubers were sampled as for the wounded tubers. Thus, all extractions of RNA, sterol and SGA were for each treatment and time point made in aliquots of the same pool of three tubers. The sampling procedure was repeated in parallel for the second tuber batch. At the specified time points, discs were frozen in liquid nitrogen and stored at −70 °C prior to extractions.

### Chemical analyses

Glycoalkaloid quantifications of cvs. Bintje and King Edward were performed by SW Seed AB (Svalöv, Sweden) using a HPLC-UV method essentially as described^18^,^48^, and according to the Swedish National Food Agency standards NMKL 13.4. Corresponding analyses of transgenic plants were made based on a slightly modified setup^49^, and using solamargine as an added internal standard^10^. Chlorophyll analysis was according to Bruinsma^50^.

Sterols were isolated from young leaves and tubers, and analysed using GC-FID as described^51^. Sterol identity and peak purity was confirmed by GC/MS analysis of selected samples and comparison to authentic sterol standards (Supplementary Table S13), and the corresponding chemical structures are illustrated in Supplementary Fig. S3. Sterols in experiments with transgenic plants were quantified by GC-MS using desmosterol, cycloeucalenol and 24-methylene cycloartanol as added internal standards for different sterol fractions^13^.

### Microarray analysis

Total RNA was extracted from tuber discs as described^52^. Transcript profiling was made using the potato 10 Kv4 cDNA microarray, developed by The Institute for Genomic Research (TIGR). This array contained 15264 cDNA clones spotted as random duplicates, 11412 of which had been validated by re-sequencing. All screening procedures were performed by the TIGR Solanaceae Expression Profiling Service, and relevant protocols are available at http://www.jcvi.org/potato/sol_ma_protocol.shtml. The effect of wounding or light exposure treatment was investigated using a common reference design, where RNA from the indicated time points was hybridized to a common reference at time zero. Each treatment was made in biological duplicates. Microarray slides were scanned with a GenePix array scanner at 532 and 635 nm. GenePix Pro.5.1 was used to qualify spot intensity, spot boundaries, and to compute spot and background intensities and their ratio. Raw data and experimental details can be found at ftp://ftp.tigr.org/pub/data/s_tuberosum/SGED/110_Folke.

Microarray raw data were analysed using software within The Linnaeus Centre of Bioinformatics warehouse (http://www.lcb.uu.se)^53^. Data processing and statistical analyses were performed in “R” (www.r-project.org), using the linear models for microarray data (LIMMA) package from Bioconductor (www.bioconductor.org). Following background correction, data were normalized within and between individual arrays using the print-tip lowess method^54^. Spots flagged as bad or not found were removed from the data. A significant difference in gene expression was assigned from stringent B-statistics (p < 0.0001) as calculated from the biological duplicates.

Normalized expression datasets from biological duplicates were averaged and imported into the Short Time-series Expression Miner (STEM, version 1.3.3) software^26^. Parameters differing from pre-setting values were: No normalization/add 0, filtering minimum absolute expression change set to 1.5, and cluster profile minimum correlation set to 0.70. A false discovery rate of 5% was used, and total cluster number was set to 50.

### Quantitative real-time PCR

Complementary DNA was synthesized from the same RNA preparations that were used for microarray analyses, using SuperScript^Tm^ III reverse transcriptase (Invitrogen Corp., Carlsbad, CA, USA). QPCR were performed with ABsolute^Tm^ QPCR SYBR^®^ Green Fluorescein mix, (Thermo Fisher Scientifics Inc., Surrey, UK) and analysed on an iQ5 Real-time PCR Detection system (Bio-Rad Laboratories Inc., Hercules, CA, USA). PCR conditions were 95 °C for 15 min, followed by 40 cycles of 95 °C for 10 s, and annealing/extension at 60 °C for 30 s. Relative expression values were calculated as described^55^. Quantifications were normalized to the endogenous β*-TUBULIN* expression, and non-treated (0 h) tuber samples were used as the calibrator. Primer DNA sequences are given in Supplementary Table S14.

## Additional Information

**How to cite this article**: Nahar, N. *et al*. Transcript profiling of two potato cultivars during glycoalkaloid-inducing treatments shows differential expression of genes in sterol and glycoalkaloid metabolism. *Sci. Rep.* **7**, 43268; doi: 10.1038/srep43268 (2017).

**Publisher's note:** Springer Nature remains neutral with regard to jurisdictional claims in published maps and institutional affiliations.

## Supplementary Material

Supplementary Figures

Supplementary Dataset 1

## Figures and Tables

**Table 1:** Glycoalkaloid level in wild-type potato cv.

Genotype	Glycoalkaloid level (mg kg^−1^ f.w.)	
	Leaves	Tubers
Wild-type	1370 ± 474 (4)	229 ± 13 (3)
35 S:*DWF1* clone S1	1306 ± 21 (2)	204 ± 28 (3)
35 S:*DWF1-L* clone S1	1430 ± 161 (2)	156 ± 41 (3)

Désirée, and the derived transgenic 35 S:*DWF1* and 35 S:*DWF1-L* plants. Glycoalkaloids were measured by HPLC-UV as the sum of α-solanine and α-chaconine, and using solamargine as an added internal standard. Mean value ± s.d. or range. Numbers in parenthesis indicate biological replicates.
