# Supplementary material for: Transcript profiling of two potato cultivars during glycoalkaloid-inducing treatments shows differential expression of genes in sterol and glycoalkaloid metabolism
Source: Sci Rep. 2017 Mar 3;7:43268. doi: 10.1038/srep43268 (PMC5335668; doi:10.1038/srep43268)
Supplement: Supplementary Figures [file srep43268-s1.pdf]

Supplementary information

Transcript profiling of two potato cultivars during glycoalkaloid-inducing treatments shows differential expression of genes in sterol and glycoalkaloid metabolism

Nurun Nahar[1], Erik Westerberg[1], Usman Arif[1], Alexandre Huchelmann[1], Alexandra Olarte Guasca[1], Lisa Beste[1], Kerstin Dalman[1], Paresh C. Dutta[2], Lisbeth Jonsson[3], Folke Sitbon[1]

[1]Department of Plant Biology, Uppsala BioCenter, Swedish University of Agricultural Sciences, and Linnean Centre for Plant Biology, P.O. Box 7080, 75007 Uppsala, Sweden

[2]Department of Food Science, Uppsala BioCenter, Swedish University of Agricultural Sciences, P.O. Box 7051, 75007 Uppsala, Sweden

[3]Department of Ecology, Environment and Plant Sciences, Stockholm University, 10691 Stockholm, Sweden

Corresponding author:

Folke Sitbon, Department of Plant Biology, Swedish University of Agricultural Sciences, Box 7080, 75007 Uppsala, Sweden

E-mail: folke.sitbon@.slu.se; Tel. +4618673243

Supplementary figure and table legends

Figure S1. Working model for sterol and SGA synthesis in potato.

Figure S2. General appearance of light-exposed tubers

Figure S3 . Chemical structure of sterols analysed.

Figure S4. STEM gene expression profiles.

Figure S5. Functional classification of differentially regulated genes.

Figure S6. Light induction of SGA levels and *DWF1-L* expression in potato tubers.

Figure S7. *DWF1-L1* promoter structures

Figure S8. Genes co-expressed with *DWF1-L.*

Figure S9. Gene structure of *DWF1* and *DWF1-L* in potato.

Figure S10. Alignment of DWF1 and DWF1-L amino acid sequences.

Figure S11. Phylogenetic relationships of potato DWF1 and DWF1-L proteins.

Figure S12. RT-PCR analysis of *DWF1* and *DWF1-L* expression in potato sense transformants.

Figure S13. RNA gel blot analysis of *DWF1* expression in potato *asDWF1* transformants.

Figure S14. QPCR analysis of *DWF1* and *DWF1-L* expression in antisense transformants.

Figure S15. Tuber sterol profiles in wild-type, *asDWF1* and *asDWF1-L* potato transformants.

Figure S16. Model for cholesterol biosynthesis in plants.

Table S1. Significantly regulated genes in Bintje following wounding.

Table S2. Significantly regulated genes in Bintje following light exposure.

Table S3. Significantly regulated genes in King Edward following wounding.

Table S4. Significantly regulated genes in King Edward following light exposure.

Table S5. Numbers of differentially expressed genes in Bintje and King Edward.

Table S6. STEM profiles in Bintje following wounding.

Table S7. STEM profiles in Bintje following light exposure.

Table S8. STEM profiles in King Edward following wounding.

Table S9. STEM profiles in King Edward following light exposure.

Table S10. Potato EST clones similar to *Arabidopsis* sterol genes.

Table S11. Potato EST clones co-expressed with *HMGR1/SMO1-L/DWF1-L* genes.

Table S12. Leaf sterol levels in wild-type, 35S:*DWF1* and 35S:*DWF1-L1* potato transformants.

Table S13. Sterol standards used for identification.

Table S14. Primers used for PCR.

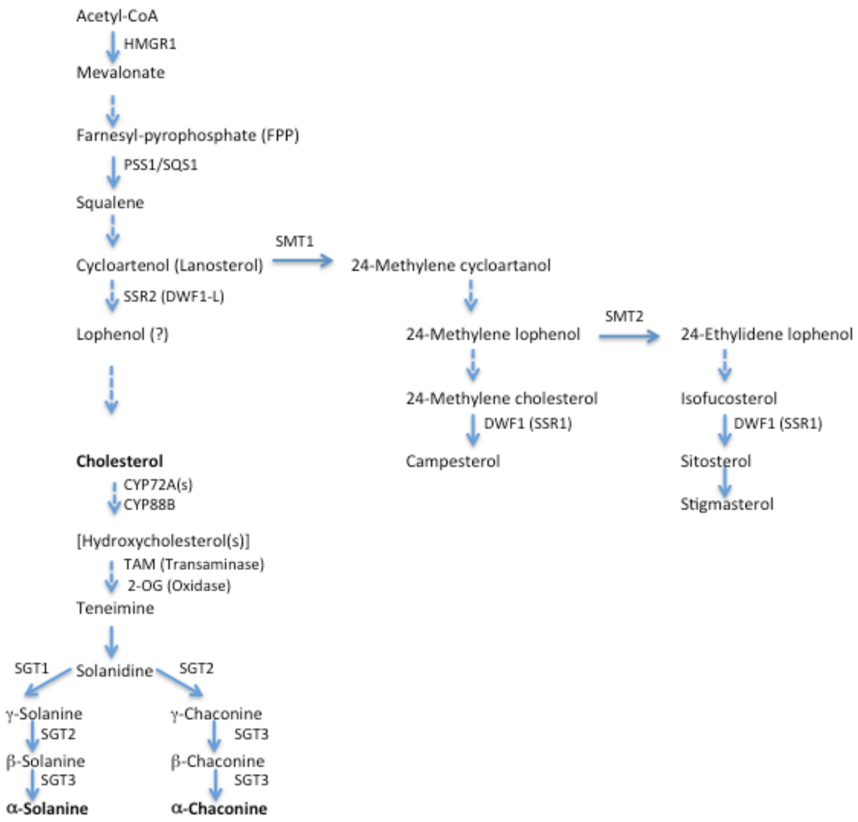

Figure S1. Working model for sterol and glycoalkaloid biosynthesis in the potato (*Solanum tuberosum*). The predominant glycoalkaloids in cultivated potato species, α-solanine and α-chaconine, are are derived from the C_{27} sterol cholesterol, and synthesized from the same aglycone, solanidine. Final conversions of solanidine to α-solanine and α-chaconine are catalyzed by three glycosyl transferases (SGT1, SGT2 and SGT3). Based on Cárdenas *et al.*[1].

Abbreviations: CYP, cytochrome P450; DWF1, sterol 24(28) reductase; HMGR, hydroxy-3-methylglutaryl CoA reductase; PSS1 (also denoted SQS1), squalene synthase; SGT1, solanidine galactosyl transferase; SGT2, solanidine glucosyl transferase; SGT3, β-solanine/β-chaconine rhamnosyl transferase; SMT1, sterol methyltransferase type 1, SMT2, sterol methyltransferase type 2; SSR2 (DWF1-L), sterol 24(25) reductase. Dashed lines indicate more than one enzymatic step.

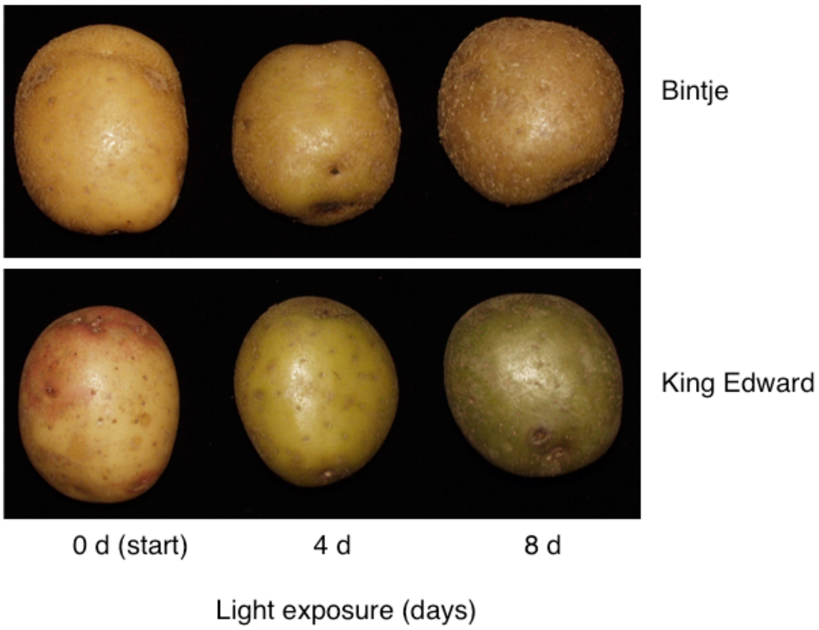

Figure S2. General appearance of Bintje and King Edward tubers subjected to light exposure. Tubers were exposed to constant white fluorescent light in a growth cabinet for the time points indicated. The transcriptional profiling and metabolic assays were performed during the initial 4 days. Spectral distribution of the light source and temperature conditions were as described[2].

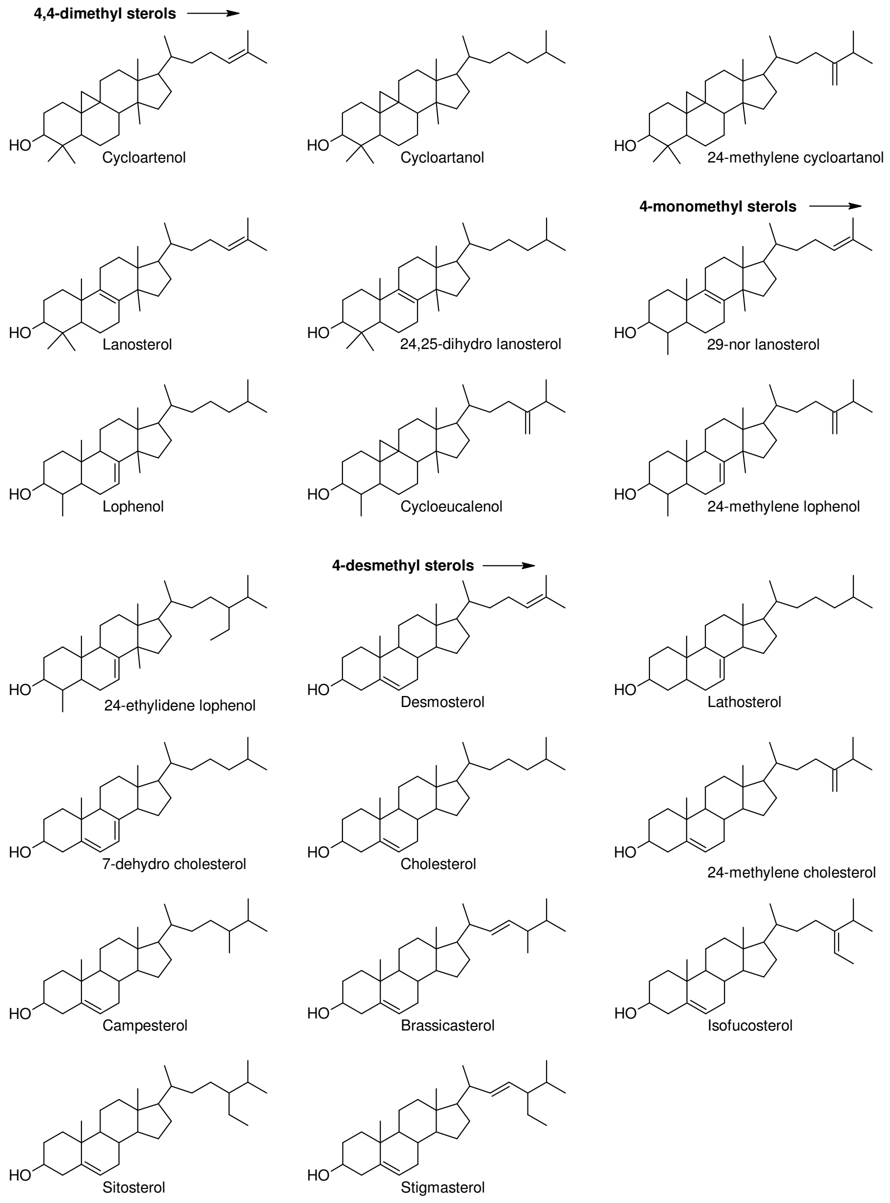

Figure S3. Chemical structure of sterols investigated. Mass to charge ratios (m/z) of molecular ions and main fragments are listed in Supplementary Table S13.

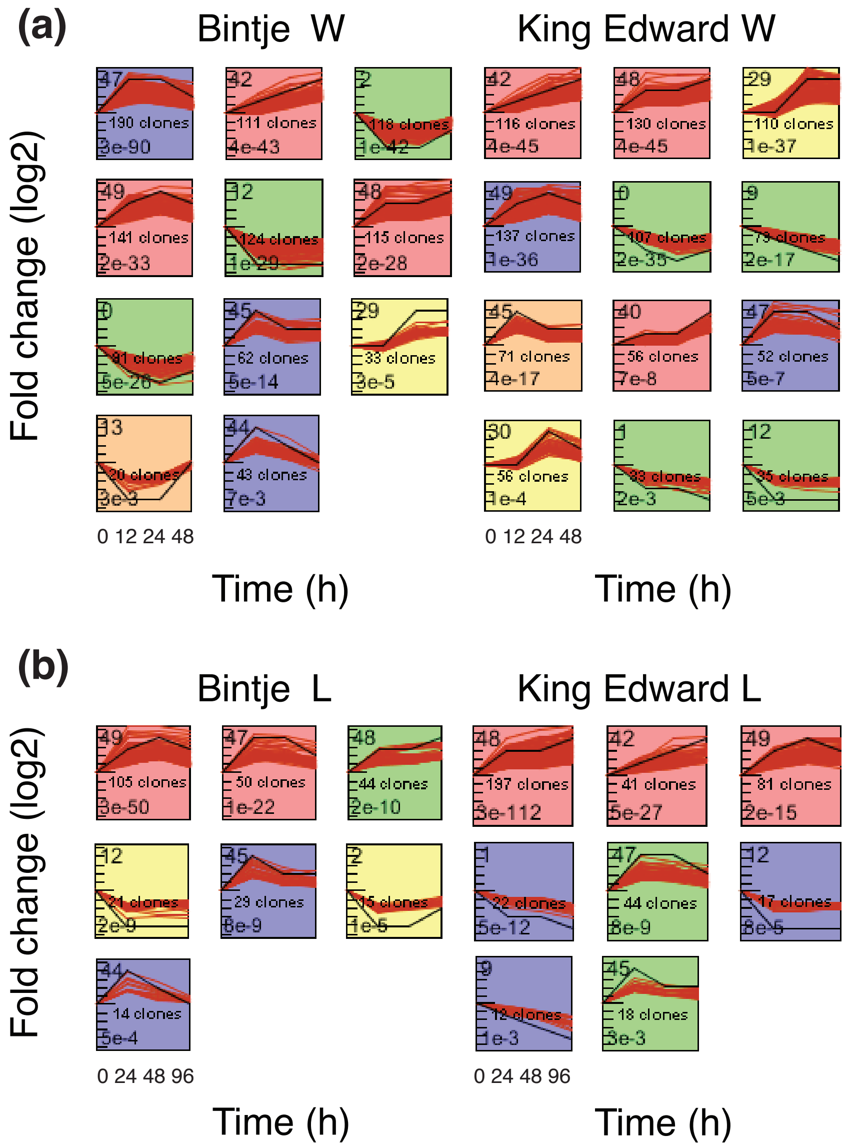

Figure S4. Temporal gene expression profiles in potato tubers subjected to a wounding stress (W) or a light exposure (L). Significant expression profiles identified by the STEM software[3] from datasets obtained by microarray analysis of potato tubers from cultivars Bintje and King Edward, subjected to wounding (**a**), or light exposure (**b**). Each profile corresponds to a specific model of temporal expression ($p<0.001$). Clusters with similar colour show similar expression pattern based on STEM correlation coefficients. The individual gene expression pattern in each cluster is indicated in red, and the specific model profile by a black bold line. For each temporal profile a profile ID number is given at the top, gene numbers in the middle, and p-values at the bottom. A full list of genes and temporal profiles is given in Supplementary Tables S6 to S9.

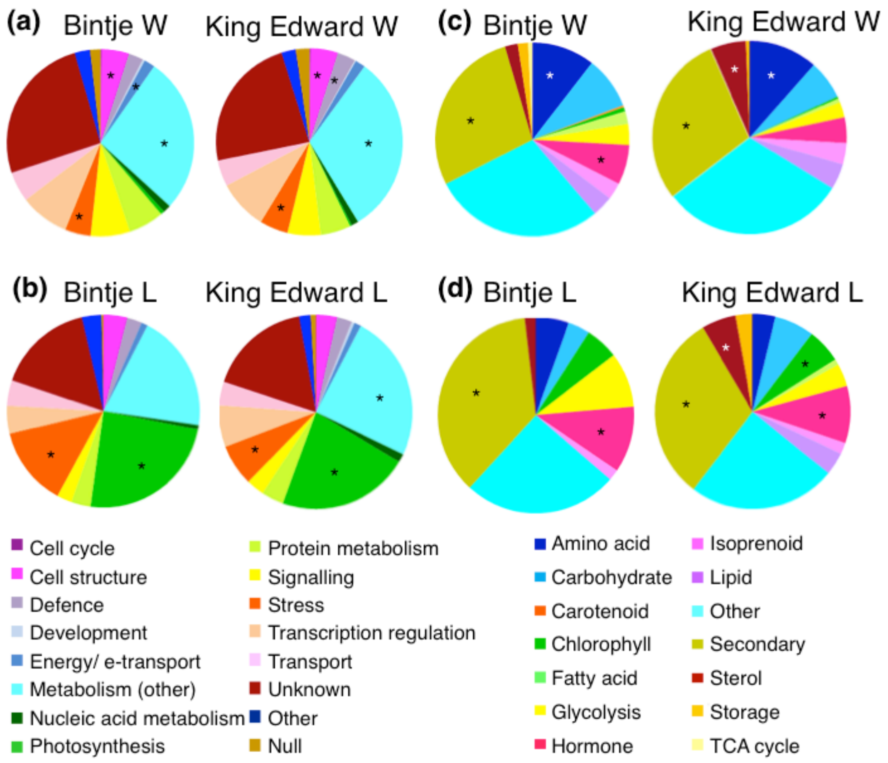

Figure S5. Functional classification of genes assigned significant temporal expression categories. (**a**) and (**b**), Gene ontology annotation categories of genes assigned significant STEM profiles (see Supplementary Fig. S4), in Bintje and King Edward tubers subjected to a wounding stress (W) or a light exposure (L). (**c**) and (**d**), The class "Metabolism" in wounded or light-exposed tubers was sub-classified into the processes indicated. Asterisks (*) indicate a statistically overrepresented class significant at $p<0.05$ (Chi-square test using Yate´s correction).

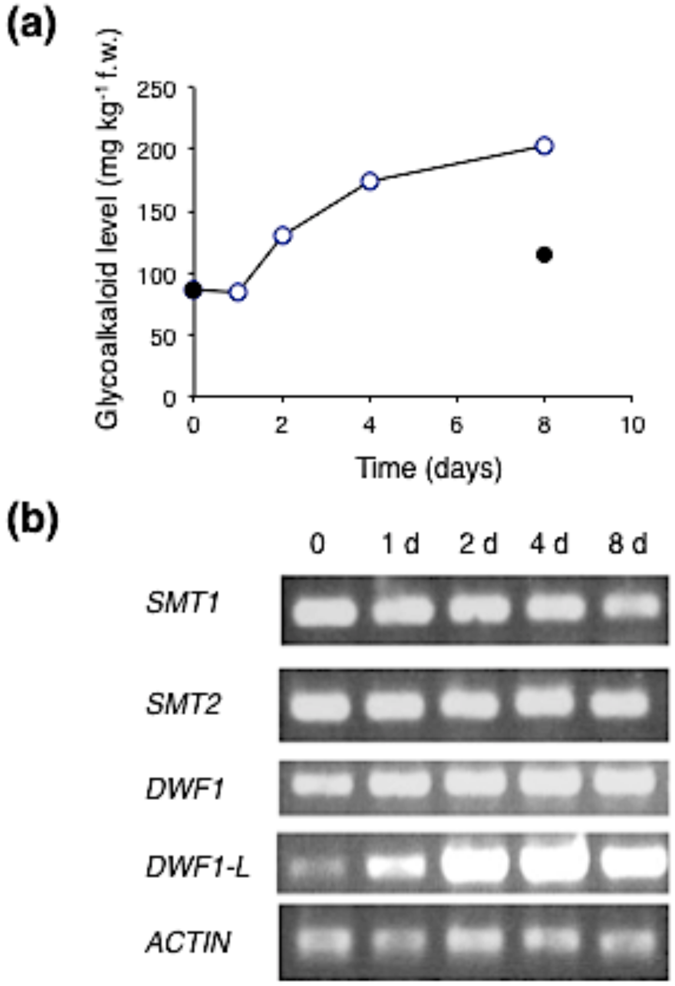

Figure S6. Glycoalkaloid levels and gene expression in potato tubers during light exposure. (**a**) Total glycoalkaloid levels were analysed by HPLC-UV in King Edward tubers exposed to white fluorescent light for the times indicated. Open symbols, light-treated; dark symbols, a parallel dark control by wrapping tubers in aluminium foil. (**b**) Semiquantitative RT-PCR analysis of RNA extracted from aliquots of the tubers analysed. Abbreviations: *SMT1*, sterol methyltransferase type 1; *SMT2*, sterol methyltransferase type 2; *DWF1*, sterol 24(28) reductase; *DWF1-L*, *DWF1*-like.

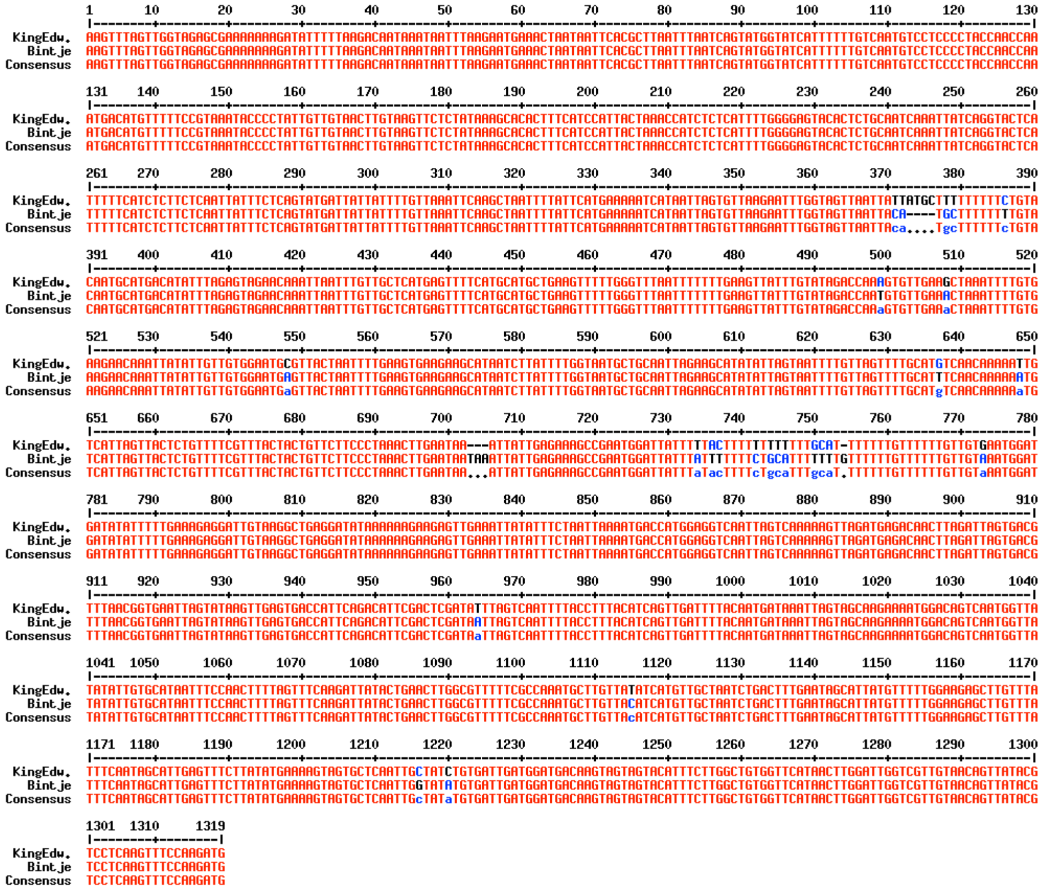

Figure S7. Comparison of upstream DNA sequences from the translational start codon in the *DWF1-L1* promoter. Promoter DNA (1300 bp) was amplified by nested PCR from genomic DNA from potato cultivars King Edward and Bintje, and sequenced in both directions. The comparison was made in MultAlin[4].

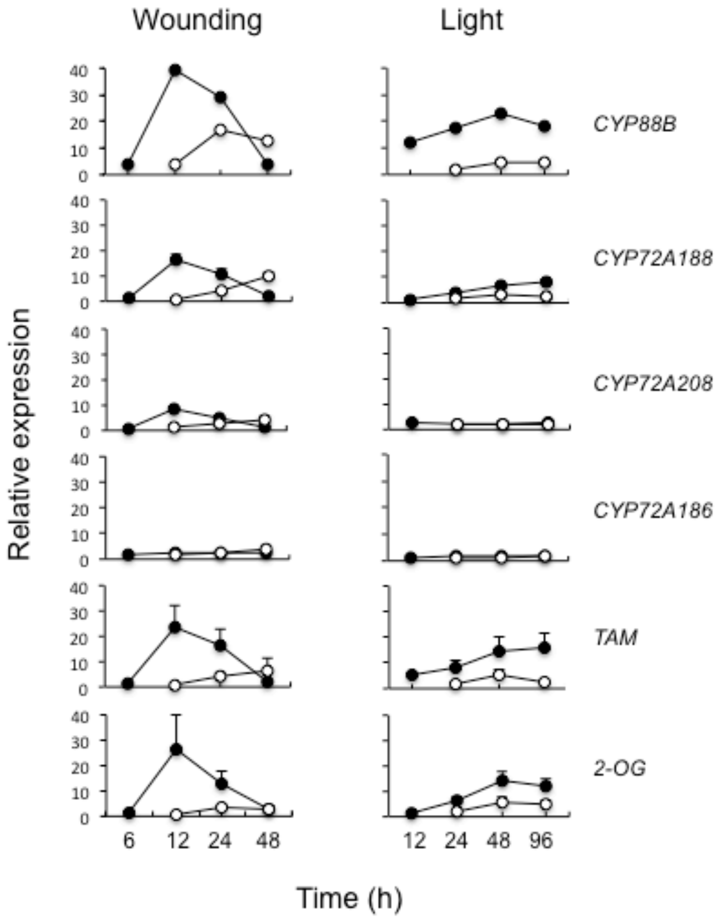

Figure S8. Temporal expression of genes coexpressed with *HMGR1/SMO1-L/DWF1-L*. Microarray analysis of potato tubers from cultivars Bintje (white circles) and King Edward (dark circles), subjected to a wounding stress (left), or a light exposure (right). Results for two additional *CYP72A* genes (*CYP72A208* and *CYP72A186*) that were not coexpressed are included for comparison. Average expression values from duplicate measurements of two biological replicates ± range or s.d. of homologous EST clones where applicable. Coexpression was judged from STEM analysis (Supplementary Fig S3 and Table S11).

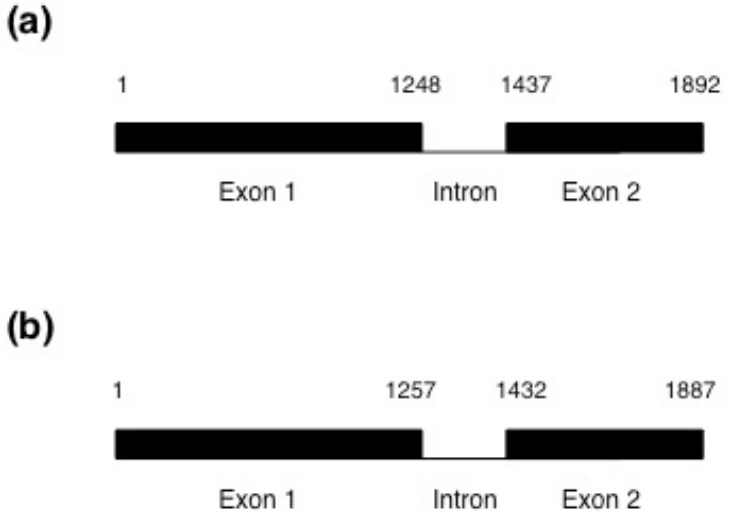

Figure S9. Genomic structure of *DWF1* and *DWF1-L*. Genomic sequences of the single *DWF1* (**a**), and *DWF1-L* (**b**), gene were retrieved from the fully sequenced genome of the wild potato species *Solanum phureja*. Intron/exon borders were deduced by comparison to fullength cDNA sequences.

```
            1                                                               60
StDWF1      MTDVQAPPR- ---PKRKKNI MDLLVQFRWI VVIFVVLPLS FLYYFSIYVG DVRSECKSYK
StDWF1-L1   MSDAKAPAAA VH-PRRKIQL VDFLLSFRWI IVIFFVLPFS FLYYFSIYLG DLKSEKKSYK
StDWF1-L2   MSDAKAPAAT VH-PRRKIQL VDFLLSFRWI IVIFFVLPFS FLYYFSIYLG DLKSEKKSYK
StDWF1-L3   MSDAKVPAAT VHHPRRKIQL VDFLLSFRWI IVIFFVLPFS FLYYFSIYLG DVKSEKKSYK
                         INS                        TMD
            61                                                             120
StDWF1      QRQKEHDENV KKVVKRLKDR NASKDGLVCT ARKPWVAVGM RNVDYKRARH FEVDLSPFRN
StDWF1-L1   QRQMEHDENV KEVVKRLEQR NAEKDGLVCT ARPPWVVVGM RNVDYKRARH FEVDLSKFRN
StDWF1-L2   QRQMEHDENV KEVVKRLGQR NAEKDGLVCT ARPPWVVFGM RNVDYKRARH FEVDLSKFRN
StDWF1-L3   QRQMEHDENV KEVVKRLEQR NAAKDGLVCT ARPPWVVVGM RNVDYKRARH FEVDLSKFRN

            121                                                            180
StDWF1      VLNIDTERMI AKVEPLVNMG QISRVTVPMN VSLAVVAELD DLTVGGLING YGIEGSSHIY
StDWF1-L1   ILDIDTERMV AKVEPLVNMG QMSRVAIPMN LSLAVLAELD DLTVGGLING FGVEGSSHIF
StDWF1-L2   ILDIDTERMV AKVEPLVNMG QMSRVTIPMN LSLAVLAELD DLTVGGLING FGVEGSSHIF
StDWF1-L3   ILDIDTERMV AKVEPLVNMG QMSRVTIPMN LSLAVLAELD DLTVGGLING FGVEGSSHIF

            181                                                            240
StDWF1      GLFSDTVVSY EVVLADGQVV RATKDNEYSD LFYAIPWSQG TLGLLVSAEI KLIPIKEYMK
StDWF1-L1   GLFSDTVVAL EVVLADGKVV RATKDNEYSD LFYAIPWSQG TLGLLVSAEI KLIPVDQYVK
StDWF1-L2   GLFSDTVVAL EVVLADGKVG RATKDNEYSD LFYAIPWSQG TLGLLVSAEI KLIPVDQYVK
StDWF1-L3   GLFSDTVVAL EVVLADGKVV RATKDNEYSD LFYAIPWSQG TLGLLVSAEI KLIPVDQYVK
                                  FAD-BD
            241                                                            300
StDWF1      LTYKPVVGNL KEIAQAYIDS FSPKDGDQDN REKVPDFVET MVYTPTEAVC MTGRYASKEE
StDWF1-L1   LTYKPVRGNL QELAQAYADS FAPKDGDQDN PSKVPEMVEG MIYGPTEGVM MTGMYASRNE
StDWF1-L2   LTYKPVRGNL KELAQAYADS FAPKDGDQDN PSKVPEMVEG MIYGPTEGVM MTGMYASKKE
StDWF1-L3   LTYKPVRGNL KELAQAYADS FAPKDGDQDN PSKVPEMVEG MIYGPTEGVM MTGMYASKKE

            301                                                            360
StDWF1      AKKKGNVINN VGWWFKTWFY QHAQTALKKG EFVEYIPTRE YYHRHTRCLY WEGKLILPFG
StDWF1-L1   AKRRGNVINN YGWWFKPWFY QHAQTALKRG EFVEYIPTRD YYHRHTRSLY WEGKLILPFG
StDWF1-L2   AKRRGNVINN YGWWFKPWFY QHAQTALKRG EFVEYIPTRD YYHRHTRSLY WEGKLILPFG
StDWF1-L3   AKRRGNVINN YGWWFKPWFY QHAQTALKRG EFVEYIPTRD YYHRHTRSLY WEGKLILPFG

            361                                                            420
StDWF1      DQWWFRFFFG WAMPPKVSLL KATQGEYIRN YYHENHVIQD MLVPLYKVGD ALEWVNREME
StDWF1-L1   DQFWFRFLLG WLMPPKIALL KATQSEAIRN YYHDHHVIQD LLVPLYKVGD CLEWVHREME
StDWF1-L2   DQFWFRFLLG WLMPPKIALL KATQSEAIRN YYHDHHVIQD LLVPLYKVGD CLEWVHREME
StDWF1-L3   DQFWFRFLLG WLMPPKIALL KATQSEAIRN YYHDHHVIQD LLVPLYKVGD CLEWVHREME

            421                                                            480
StDWF1      VYPLWLCPHR LYRLPLKTMV YPEPGFELHK RQGDTKYAQM YTDVGVYYAP GPILRGEVFD
StDWF1-L1   VYPIWLCPHR IYKLPVRPMI YPEPGFEKHK RQGDTEYAQM YTDIGVYYVP GAVLRGEPFD
StDWF1-L2   VYPIWLCPHR IYKLPVRPMI YPEPGFEKHK RQGDTEYAQM YTDVGVYYVP GAVLRGEPFD
StDWF1-L3   VYPIWLCPHR IYKLPVRPMI YPEPGFEKHK RQGDTEYAQM YTDVGVYYVP GAVLRGEPFD

            481                                                            540
StDWF1      GIEAVRKLES WLIENHGFQP QYAVSELTEK NFWRMFDGSL YENCRKKYRA IGTFMSVYYK
StDWF1-L1   GSEKCRQLEL WLIENHGFQA QYAVTELTEK NFWRMFDNSL YEQCRRKYKA IGTFMSVYYK
StDWF1-L2   GSEKCRQLEL WLIENHGFQA QYAVTELTEK NFWRMFDNSL YEQCRRKYKA IGTFMSVYYK
StDWF1-L3   GSEKCRQLEL WLIENHGFQA QYAVTELTEK NFWRMFDNSL YEQCRRKYKA IGTFMSVYYK
                                                              CaM-BD
            541
StDWF1      SKKGKKTEKE VQDAEQETAE VETPEVDEPE D
StDWF1-L1   SKKGRKTEKE VQEAEQEKAE QETPEADERA N
StDWF1-L2   SKKGRKTEKE VQEAEQEKAE QETPEADEPA N
StDWF1-L3   SKKGRKTEKE VQEAEQEKAE QETPEADEPA N
```

Figure S10. Alignment of deduced potato DWF1 and DWF1-L proteins. Nucleotide sequences were obtained from Kennebec cDNA clones, and alignments of deduced aa sequences were made using MultAlin[4]. Amino acid residues with a high degree of conservation (>90 %) are labelled in red, and residues with a low conservation are labelled in blue/black. Indicated in the alignment is a stretch of inserted hydrophobic residues (INS) that is characteristic for the DWF1-like (DWF1-L) type of proteins. Also displayed are conserved domains in plant DWF1 proteins, including a suggested transmembrane domain (TMD)[5], a FAD-binding domain (FAD-BD)[6], and a Calmodulin-binding domain (CaM-BD)[7]. EMBL nucleotide database acc. no: StDWF1 (FN995649), StDWF1-L1 (FN995650), StDWF1-L2 (FN995651), StDWF1-L3 (FN995652).

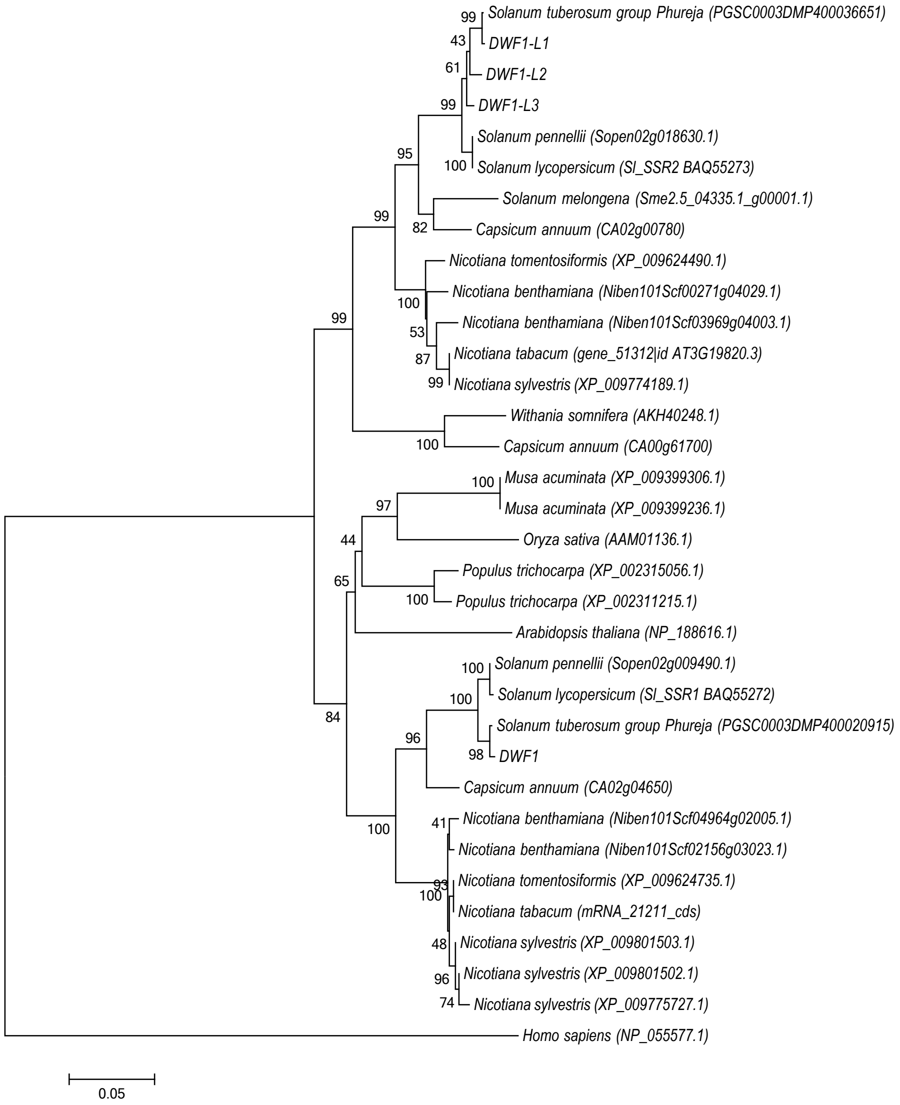

Figure S11. **Phylogenetic relationships of potato DWF1 and DWF-L proteins.** A bootstrap consensus neighbour-joining tree was constructed using ClustalW and MEGA6[8], with default settings and a number of 1000 replications. The human (*Homo sapiens*) orthologue DHCR24 was included as an outgroup. The tree was made using 34 amino acid sequences deduced from available full-length cDNA sequence information (NCBI and Sol Genomic Network databases), and with an emphasis on Solanaceous plant species. All positions containing gaps and missing data were eliminated. There were a total of 504 positions in the final dataset. Numbers at branches indicate bootstrap support.

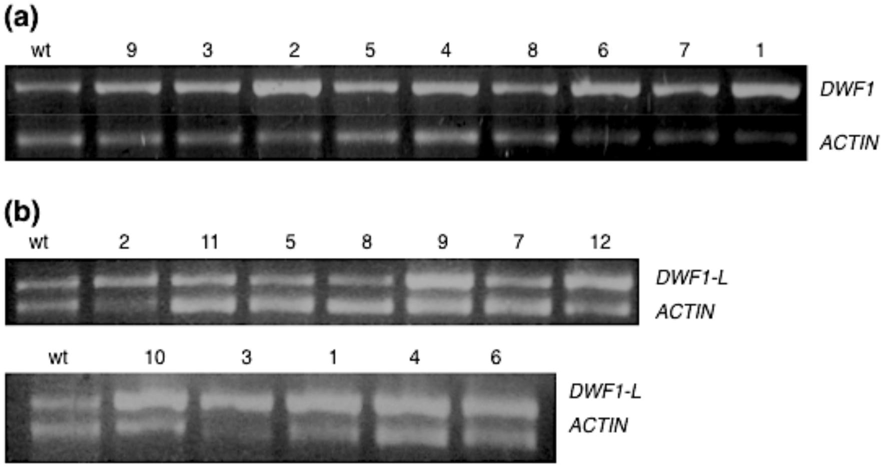

Figure S12. Multiplex RT-PCR analysis of transgene expression. Expression of *DWF1* and *DWF1-L* was assayed using multiplex RT-PCR in leaves from wild-type potato Désirée plants, and derived 35S:*DWF1* (**a**) and 35S:*DWF1-L* (**b**) transformants. An *ACTIN-101* primer pair was used as an internal control for amplification. Transformant clone numbers correspond to those in Supplementary Table S12.

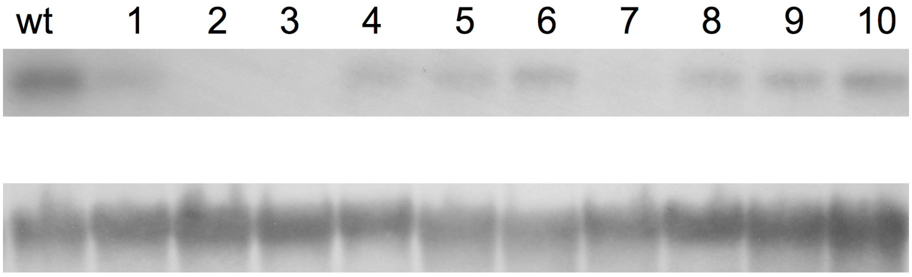

Figure S13. RNA gel blot analysis of as*DWF1* potato transformants. Total RNA was extracted from young leaves of a wild-type Désirée plant and ten derived antisense as*DWF1* transformants, and 20 µg was transferred onto a nylon membrane and hybridized under stringent conditions with a 500 bp 5′-terminal *DWF1* probe (upper), and as a loading control washed and re-hybridized with a *16S rRNA* probe (lower).

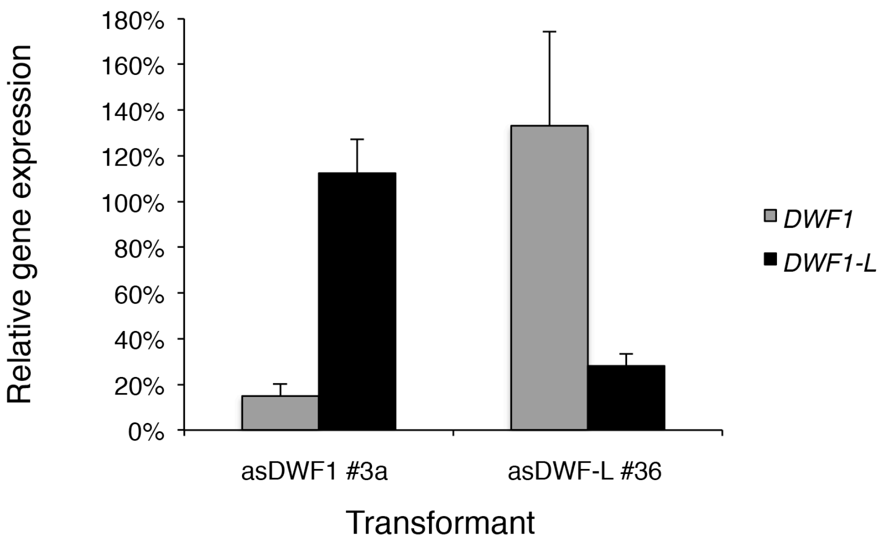

Figure S14. Expression of *DWF1* and *DWF1-L* genes in *asDWF1* and *asDWF1-L* potato transformants. Gene expression was monitored by QPCR analyses of RNA extracted from young leaves of wild-type and antisense transgenic plants that were grown at three separate occasions during a period of one year. Expression values were normalised to the expression of the *β-TUBULIN* gene*,* and to the corresponding normalised expression in wild-type plants grown in parallel. For each genotype, a similar expression profile was obtained from at least one independent transformant. Mean value ± s.d. of three plants each analysed in technical triplicates.

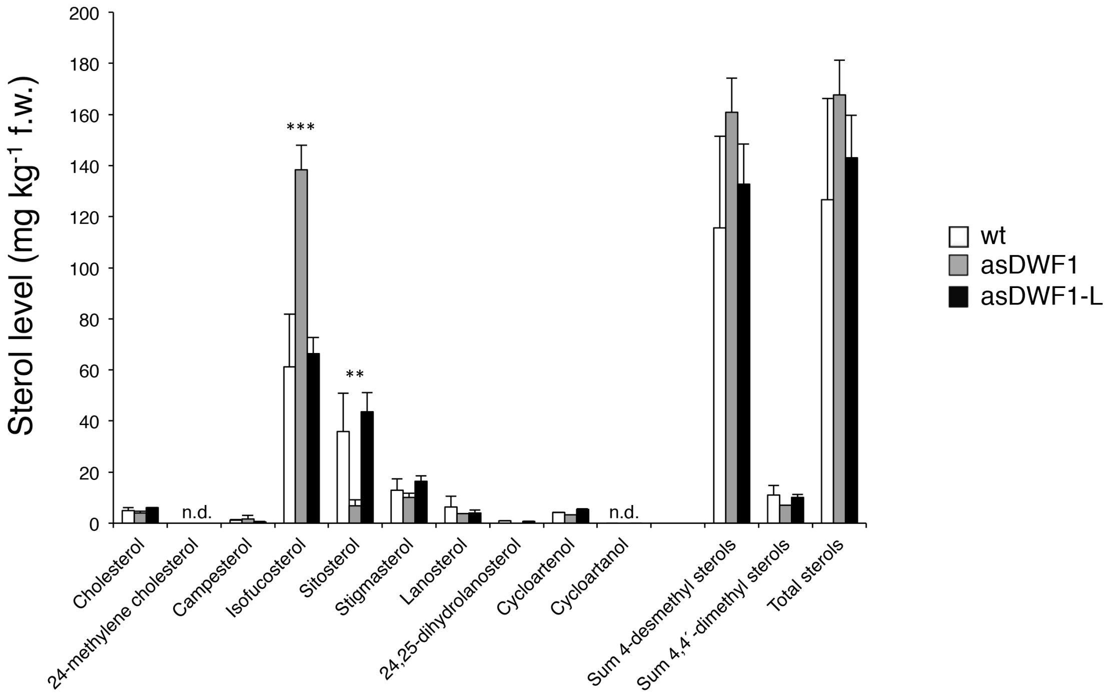

Figure S15. Sterol profile in tubers from wild-type potato plants and antisense *asDWF1* and *asDWF1-L* transformants. The 4-desmethyl and 4,4 dimethylsterol levels were analysed by GC-MS. A difference from wild-type significant at p<0.01 (**) or p<0.001 (***) is indicated by asterisks. Mean value ± s.d. for plants grown at separate occasions during a period of at least one year. Wild-type (n=5), *asDWF1* line #3a (n=4) and *asDWF1-L* #36 (n=2). For all genotypes, the sum of 4-monomethyl sterols was below 5 mg kg^{-1}, and are not shown for clarity.

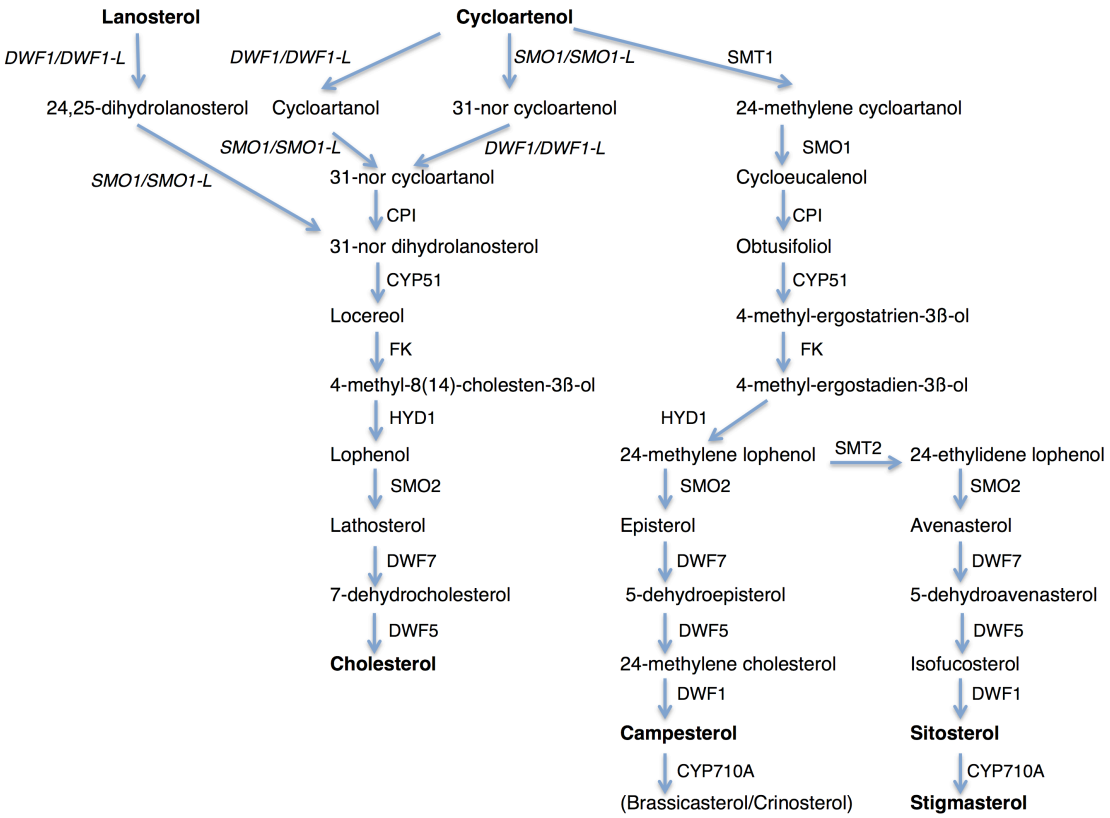

Figure S16. Model for the biosynthesis of cholesterol and other sterols in plants. Each arrow indicates a catalytic step. The metabolic steps at the dimetylsterol level, putatively catalysed by SMO1/SMO1-L and DWF1/DWF1-L, are indicated in italics. Occurrence of DWF1-L is seemingly specific to plant species containing steroidal glycoalkaloids, other plant species likely utilise DWF1 also at the 4,4-dimethyl sterol level. Major endpoint 4-desmethyl sterols in potato are indicated in bold. Abbreviations: DWF1-L, sterol 24(28) reductase-like; SMT1, sterol methyltransferase type 1; SMO1-L, sterol C4-methyl oxidase type 1-like; SMO1, sterol C4-methyl oxidase type 1; CPI, cyclopropyl sterol isomerase; CYP51, sterol 14-demethylase; FK, sterol Δ^{14}-reductase; HYD1, Δ^{8}-Δ^{7} sterol isomerase; SMT2, sterol methyltransferase type 2; SMO2, sterol C4-methyl oxidase type 2; DWF5, sterol Δ^{7}-reductase; DWF7, sterol Δ5-reductase; DWF1, sterol 24(28) reductase; CYP710A, sterol C-22 desaturase.

Supplementary References

1. Cárdenas, P.D. *et al.* The bitter side of nightshades: Genomics drives discovery in *Solanaceae* steroidal alkaloid metabolism. *Phytochemistry* **113,** 24-32 (2014).
2. Petersson, E.V**.** *et al.* Glycoalkaloid and calystegine levels in table potato cultivars subjected to wounding, light, and heat treatments. *J. Agric. Food Chem.* **61,** 5893-5902 (2013).
3. Ernst, J. & Bar-Joseph, Z. STEM: a tool for the analysis of short time series gene expression data. *BMC Bioinformatics* **7,** 191-201 (2006).
4. Corpet, F. Multiple sequence alignment with hierarchical clustering. *Nucl. Acids Res.* **16,** 10881-10890 (1988).
5. Klahre, U. *et al.* The Arabidopsis DIMINUTO/DWARF1 gene encodes a protein involved in steroid synthesis. *Plant Cell* **10,** 1677-1690 (1998).
6. Du, L. & Poovaiah B.W. Ca^{2+}/calmodulin is critical for brassinosteroid biosynthesis and plant growth. *Nature* **437,** 741-744 (2005).
7. Mushegian, A.R. & Koonin, E.V. A putative FAD-binding domain in a distinct group of oxidases including a protein involved in plant development. *Protein Science* **4,** 1243-1244 (1995).
8. Tamura, K., Stecher, G., Peterson, D., Filipski, A. & Kumar, S. MEGA6: Molecular evolutionary genetics analysis version 6.0. *Mol. Biol. Evol.* **30,** 2725-2729 (2013).
